# Supplementary material for: Simple approach for ranking structure determining residues
Source: PeerJ. 2016 Jun 22;4:e2136. doi: 10.7717/peerj.2136 (PMC4924125; doi:10.7717/peerj.2136)
Supplement: File S4 — It is shown the [b]RMSD, RMSF, and the hierarchy position of each residue in two different conditions. In Figure R1, we show the differences between the complex estrogen receptor bound with DNA after 25 ns molecular dynamics simulation and the estrogen receptor unbound from DNA after 50 ns simulation. In Figure R2, we show the convergence between simulating 25 ns and extending simulation to 50 ns of 6aJL2. [file peerj-04-2136-s004.docx]

1- Human receptor estrogen alpha (1HCQ PDB code) is in complex with DNA in MD simulation. Dose the experiment done in complex with DNA, too? It would be more informative if you could compare the crucial residues in this protein in the presence and absence of DNA.

Experiments detailed in Ref. Deegan *et al.,* 2010, where DNA was bound to the estrogen receptor and some of its mutants, concluded that mutants decreased the DNA recognition by estrogen receptor. To compare the crucial residues in the absence of DNA bound to the receptor, we performed a 50 ns molecular dynamics simulation under same conditions detailed in Materials and Methods. Results showed that the unbound dimer has different motion from motions of DNA-bound dimer, as shown in RMSD and RMSF plots (Fig. R1A and Fig. R1B, respectively). By comparing normalized theoretical scores of the residues between these two simulations, there are notable changes on the contribution of each residue into the dimer (Fig. R1C).

**B**

**A**


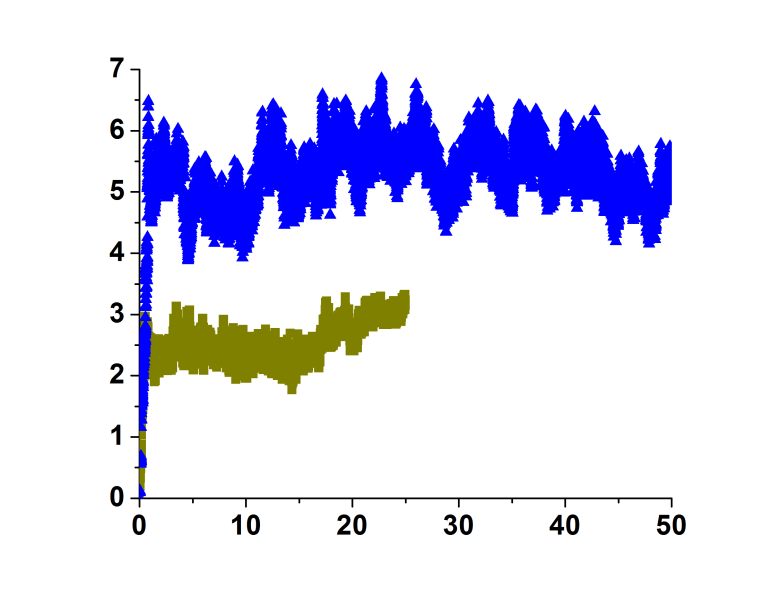


**RMSD (Å)**

**Time (ns)**


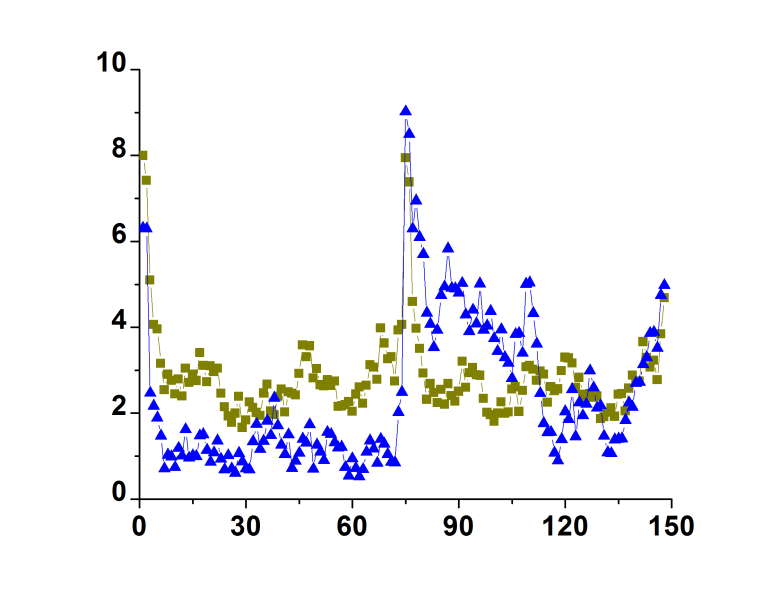


**RMSF (Å)**

**Residue**

**C**


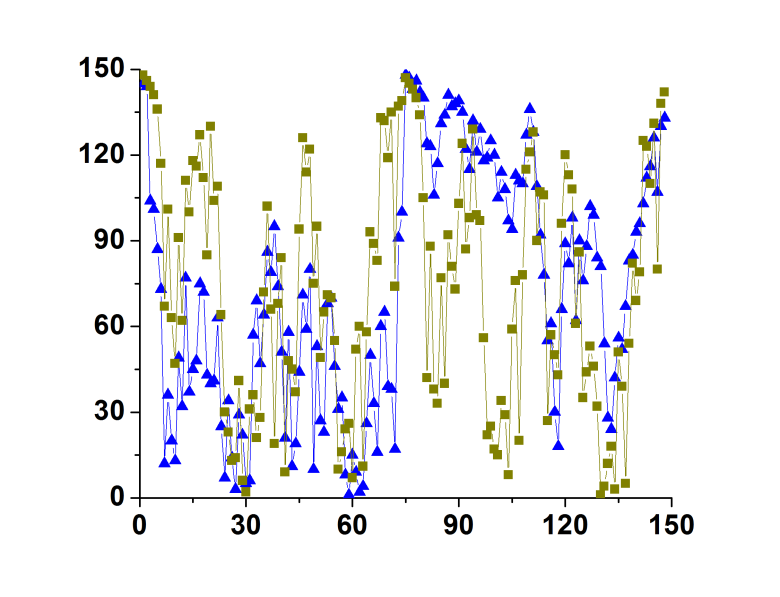


**Hierarchy position**

**Residue**

**Fig. R1. Comparison between free estrogen dimer and complexed with DNA**. In yellow is shown the difference of the complex estrogen receptor bound with DNA after 25 ns molecular dynamics simulation; and in blue, the estrogen receptor unbound from DNA after 50 ns simulation. **A.** RMSD difference. **B.** RMSF values of each residue. **C.** Hierarchy position of each residue.

All the results of simulating estrogen receptor bound to DNA generated data which were remarkably different from the results obtained for the dimer unbound to DNA. However, as stated in the text, we cannot predict which residues are going to recognize precisely interaction with its respective ligand.

2- Even though RMSD of most proteins show equilibrated state by 25ns simulation time, however it is still not enough time to consider as equilibration. I recommend authors select one of the proteins and continue the simulation for at least another 25ns and compare it with non extended simulation.

We extended 6aJL2 (PDB 2W0K) simulation and compared theoretical scores as shown below. Trajectory maintained the same RMSD difference (Fig. R2A). Slight, but not significant changes could be detected in RMSF values (Fig. R2B). Also, theoretical scores as well as hierarchy were not dramatically affected by extending another 25 ns (Fig. R2C).

**RMSD (Å)**

**Time (ns)**

**A**


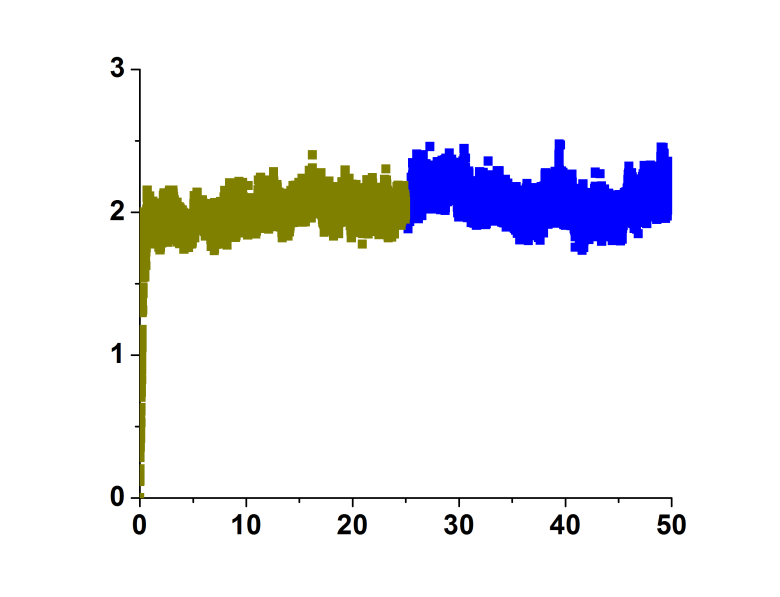

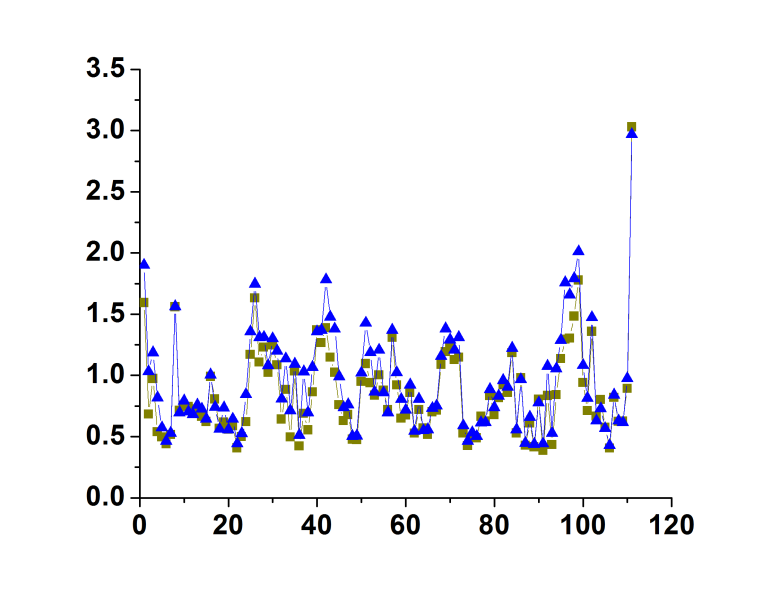


**B**

**RMSF (Å)**

**Residue**


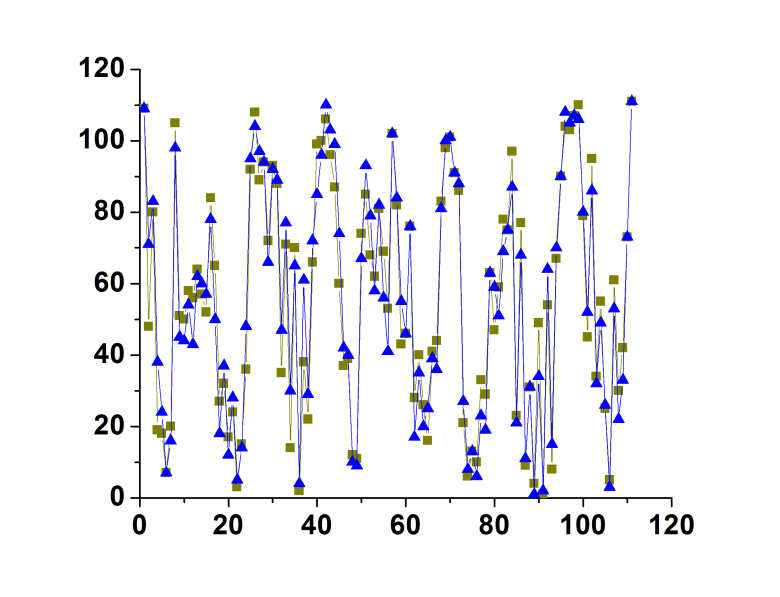


**Hierarchy position**

**Residue**

**C**

**Fig. R2. Comparison between molecular time simulations**. In yellow is shown the structure 6aJL2 after 25 ns molecular dynamics simulation; in blue, 6aJL2after extending simulation to 50 ns simulation. **A.** RMSD difference. **B.** RMSF values of each residue. **C.** Hierarchy position of each residue.

By extending simulation time 25 ns more, hierarchy position of each residue was not considerably modified.
