# Supplementary figures and images for: Simple approach for ranking structure determining residues
Source: PeerJ. 2016 Jun 22;4:e2136. doi: 10.7717/peerj.2136 (PMC4924125; doi:10.7717/peerj.2136)

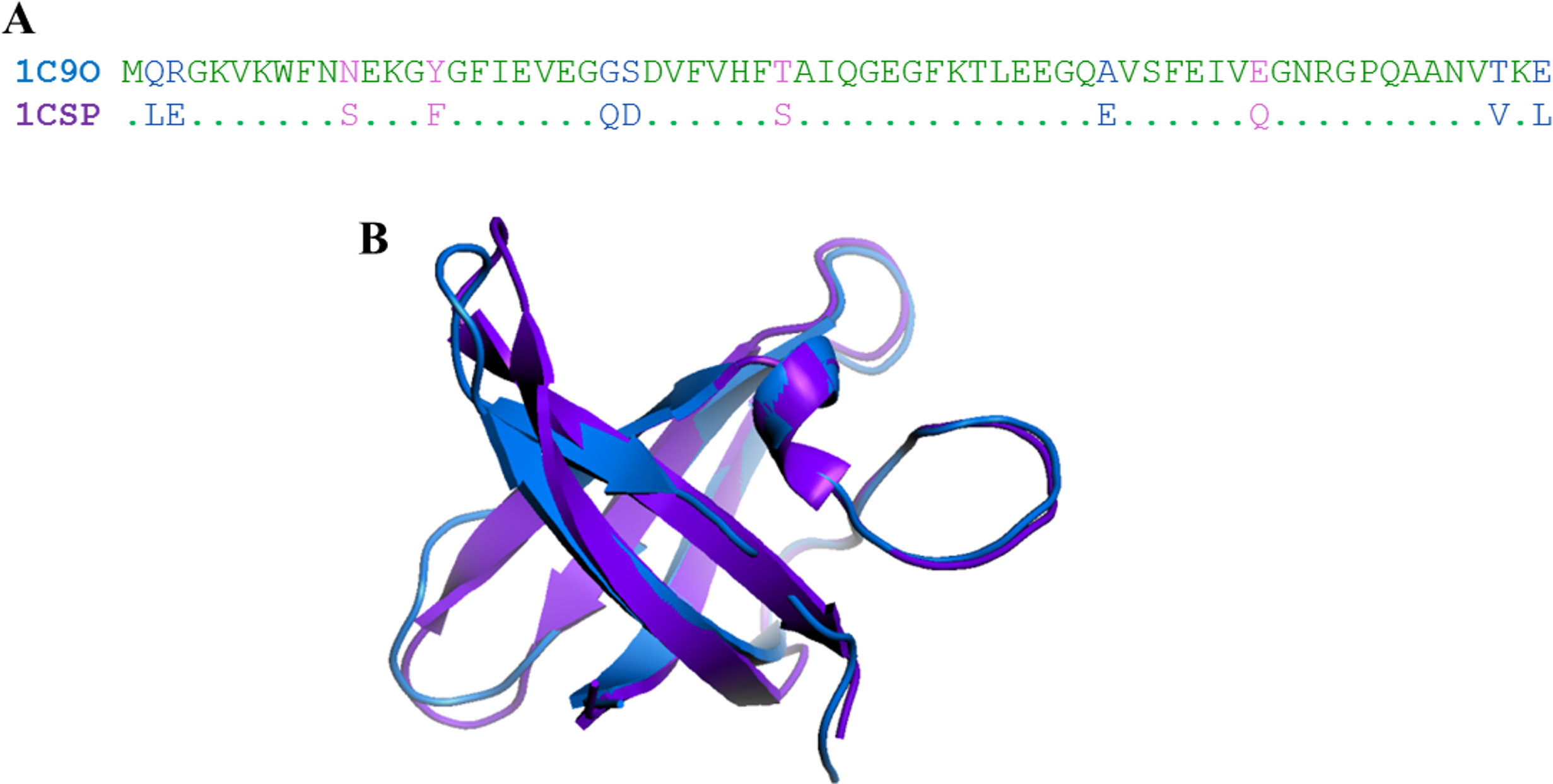

Supplement: Figure S1 — (A) Sequence aligning between cold shock proteins from B. caldolyticus (PDB-1C9O) and B. subtilis (PDB-1CSP). In green are remarked identical residues; in pink, similar residues; and in blue, sequence mismatch. (B) Despite that having different thermodynamic parameters, overlapping cold shock proteins shows high structural homology with a main chain RMSD of 0.5 Å calculated with PyMOL software. [file peerj-04-2136-s005.png]

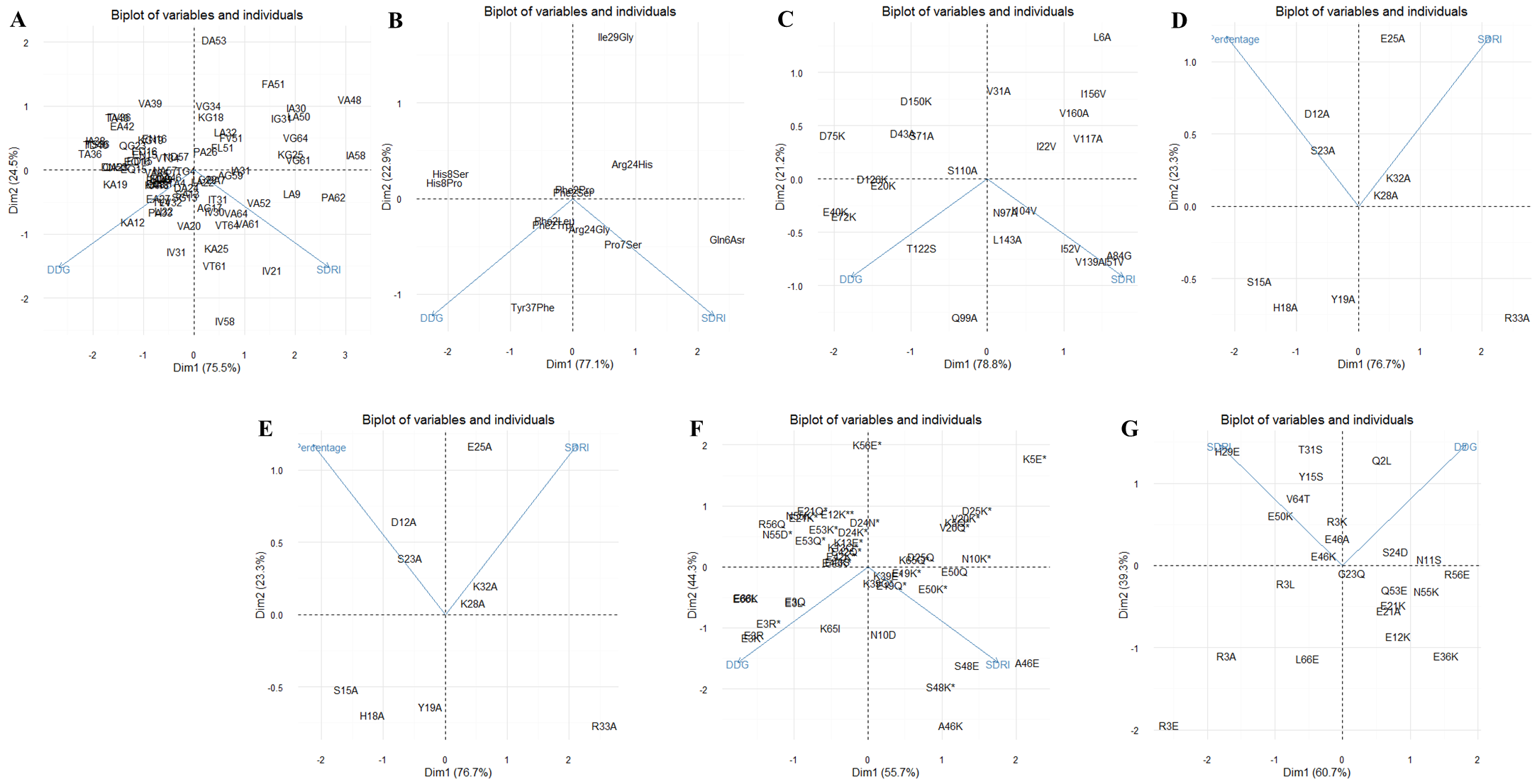

Supplement: Figure S2 — Detailed information of circular biplots indicating the position of each mutation of its respective protein is shown. Variables are shown as blue vectors, in Dim1 was measured SDRI scores and in Dim2 thermodynamic data. (A) Chymotrypsin inhibitor (PDB entry 2CI2); (B) 6aJL2 (PDB entry 2W0 K); (C) apoflavodoxin (PDB entry 1FTG); (D) arc repressor (PDB entry 1ARR); (E) DNA-binding domain of the estrogen receptor α (PDB entry 1HCQ); (F) cold shock protein from B. subtilis; (G) cold shock protein from B. caldolyticus; and (H) the JAK interaction region of SOCS5 (PDB entry 2N34). [file peerj-04-2136-s006.png]
